# The Effect of Age on Outcome in Excision of Chronic Osteomyelitis with Free Muscle Flap Reconstruction

**DOI:** 10.7150/jbji.31764

**Published:** 2019-08-02

**Authors:** John Victor Kendall, Martin McNally, Christopher Taylor, Jamie Ferguson, Svetlana Galitzine, Paul Critchley, Henk Giele, Alexander John Ramsden

**Affiliations:** 1 Severn Deanery (Bristol), UK - Trauma & Orthopaedic Registrar; 2 The Bone Infection Unit, Nuffield Orthopaedic Centre, Oxford University Hospitals, Oxford, UK; 3 Derriford Hospital, Plymouth, UK

**Keywords:** Osteomyelitis, free flap, free tissue transfer, old age, elderly, bone infection, fracture-related infection

## Abstract

**Introduction:** Curative surgical treatment of chronic osteomyelitis often requires free tissue transfer if there is significant soft tissue compromise. We investigated whether age influenced outcomes of curative osteomyelitis excision in those patients requiring free muscle flap soft tissue reconstruction.

**Methods:** We assessed ninety-five consecutive patients treated with excision of chronic osteomyelitis, skeletal stabilisation/reconstruction and free muscle transfer between 2006 and 2012. We compared outcomes of those aged ≥60 years (*n*=23) with those <60 years old (*n*=72).

**Results:** Groups were similar with regard to Cierny and Mader anatomic type and physiological host classification. Length of procedure and length of hospital stay were similar for both groups. There was a greater proportion of ASA grade III patients in the older cohort. Infection recurrence occurred in one of the older cohort (4.3%) and in seven patients in the younger cohort (9.9%) at a mean follow-up of 42 months (range 11-131 months), this was not statistically significant (*p*=0.27874). There were five free flap losses (6.9%) in the younger group and none in the older group. A greater proportion of patients from the younger cohort required further unplanned surgery (28%) compared to the older group (4.3%), which was statistically significant (*p*=0.01174). Seven patients (7.4%) had serious medical complications - five of whom were in the younger cohort, including one mortality.

**Conclusions:** Both the young and old can enjoy satisfactory outcomes from surgical resection of chronic osteomyelitis with simultaneous orthoplastic reconstruction including free tissue transfer. Age alone should not be a barrier to potentially curative surgical treatment.

## Introduction

Chronic osteomyelitis is a debilitating condition associated with bone necrosis, soft tissue disruption and pain resulting in significant morbidity and loss of function. There is frequently a significant soft tissue component to the pathology. Chronic osteomyelitis in the developed world is most commonly the result of contiguous spread e.g. from open fractures, soft tissue ulceration, or from fracture fixation. Despite improvements in surgical technique, implant design and infection prophylaxis, the incidence of osteomyelitis is increasing^1^. It represents a significant complication of current orthopaedic practice and the legacy of injuries sustained decades before.

Treatment requires a multi-disciplinary approach. Curative treatment involves complete excision of necrotic bone and compromised soft tissues^2^, adequate representative sampling and diagnosis of the causative organism^3^, ablation of dead space, skeletal stabilisation/reconstruction, reconstruction of soft tissue defects with vascularised tissue and targeted post-operative antibiotic therapy. Reconstruction may require complex combined orthoplastic procedures using techniques such as Ilizarov bone transport and microvascular free tissue transfer. Microvascular reconstruction in this group is perceived as challenging due to soft tissue and perivascular fibrosis, previous axial vessel injury, vascular spasm and difficult access due to external fixators.

Age may also be considered a factor in determining a patient's suitability for a long surgical procedure^4^. Cierny and Mader^5^ identified host factors adversely affecting prognosis in the treatment of chronic osteomyelitis, which included advanced age. Consequently, in elderly patients with chronic osteomyelitis amputation may be considered rather than attempting limb salvage and complex reconstruction^6^. The ideal setting for management of chronic osteomyelitis is a dedicated unit where appropriate multi-disciplinary treatment can be provided. In this environment high long-term cure rates can be achieved^2^, ^7^-^8^.

Free tissue transfer is a powerful reconstructive tool that allows import of healthy tissue into heavily scarred areas. In our institution, the standard reconstruction technique for patients with chronic osteomyelitis and significant soft tissue compromise involves a single-stage approach with free tissue transfer immediately after osteomyelitis excision and orthopaedic reconstruction. We routinely use free muscle flaps covered with unmeshed split-thickness skin graft as they are reliable, have acceptable donor site morbidity, contour well and due to their high vascularity, can aid osseous healing and deliver parenteral antibiotics to the affected site.

Klein *et al.*^9^ showed that microvascular reconstruction, predominantly for head and neck defects, was a viable reconstructive option in a patient cohort with a minimum age of 78 years. Sosin *et al.*^10^ showed that mortality and catastrophic flap complications were not increased by age in patients >70 years old who required free tissue scalp reconstruction. However, the head and neck has a rich vascular network. We were interested to see if similar conclusions could be reached in curative orthoplastic surgery for chronic osteomyelitis, where vessels often lie in an area of previous trauma that has been subjected to long-standing infection. Is curative surgery less successful and are the rates of medical and surgical complications higher in elderly patients undergoing excision of chronic osteomyelitis with simultaneous orthoplastic reconstruction including free soft tissue transfer?

## Methods

All patients who underwent chronic osteomyelitis excision with simultaneous reconstruction involving free tissue transfer between 2006 and 2012 were included. All patients had confirmed chronic osteomyelitis as defined as having symptoms for at least six months with clinical and radiological features accompanied by at least one of the following: the presence of a sinus, an abscess or intra-operative pus, supportive histology, or two or more microbiological cultures with indistinguishable organisms^11^. When microbiological cultures were negative, a patient was only included when there was positive histology, a draining sinus or intra-operative pus.

Demographic, disease, surgical and outcome data was entered prospectively into the electronic patient record system. The hospital health informatics department provided data on date and length of hospital stay and length of procedure. All data was collated for statistical analysis. Table 1 summarises the data fields collected.

All patients were managed within a specialist bone infection unit under the joint care of an orthopaedic surgeon, a plastic surgeon and an infectious diseases physician. All patients were managed using a previously described protocol based on the principles of osseous debridement, representative intra-operative sampling, skeletal stabilisation, dead space management and immediate soft tissue reconstruction^7^. Following surgery, patients received between 6 and 12 weeks of antibiotic therapy - initially broad-spectrum, then rationalised based on identified organisms and sensitivities from intra-operative sampling.

Patients were classified using the Cierny-Mader (C-M) staging system^5^ at the time of surgery and the ASA classification system for pre-operative anaesthetic risk assessment^12^. Cierny and Mader categorised patients with osteomyelitis based on anatomic classification (Type I: medullary, Type II: superficial cortical, Type III: localised cortical and medullary, Type IV: segmental/diffuse) and based on physiologic host classification (Group A: healthy, Group B: compromised host, Group C: severely compromised host/treatment deemed worse than the disease) giving an overall stage. Group B hosts can have systemic compromise (Bs) or local compromise (Bl). Table 2 illustrates the range of compromise found in these patients, which can have a major effect on outcome.

The definition of 'elderly' is controversial. We used the definition for older persons adopted by the United Nations in their World Population Aging 2013 publication^13^. We divided our patients into those <60 years old and those ≥60 years. The primary outcome was eradication of infection at final follow-up. We defined treatment failure as: (1) Infection recurrence with positive cultures from radiologically-guided aspiration or biopsy; (2) Recurrent sinus formation; (3) Further surgery performed for infection as defined above; (4) Any case requiring long-term suppressive antibiotic therapy for persistent symptoms; (5) Related amputation (i.e. failure of limb salvage). Secondary outcomes were free flap failure, length of procedure, length of hospital stay, medical and surgical complications and need for further operative procedures.

### Statistical Analysis

Data was analysed using Prism 7 (GraphPad Software, Inc.). All data were considered to be non-parametric. Associations between categorical variables were made using Fisher's Exact test on account of small sample size (http://www.quantitativeskills.com/sisa/statistics/fisher.htm). Continuous variables were compared using an unpaired, two-tailed Mann-Whitney U test. A *p*-value of <0.05 was considered statistically significant.

## Results

We identified 95 consecutive patients with chronic osteomyelitis requiring radical excision of necrotic bone and compromised soft tissues with free flap reconstruction. The distribution of osteomyelitis by bone involvement is shown in Table 3.

The free flap reconstruction used was a free gracilis flap in eighty patients (84%) and a free latissimus dorsi flap in eleven (12%). The remaining four cases used a rectus abdominis flap, a vastus lateralis flap, a fibula flap and a lateral thigh flap.

Seventy-two patients were under 60 years of age with a mean age of 42 years (range 18-59 years). Sixty-six patients (92%) had chronic infection of the tibia. Fifty-seven patients (82%) were reconstructed with a free gracilis flap and nine (13%) with a latissimus dorsi flap.

**Figure 1:**
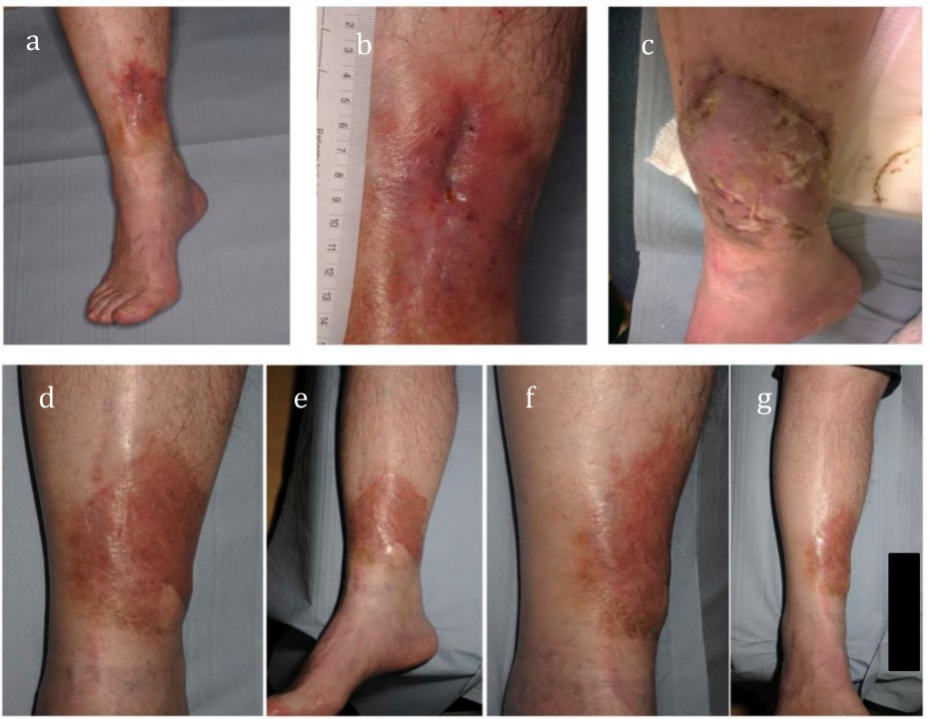
A patient from the older cohort with C-M Stage III Bls osteomyelitis of the tibia with a sinus **(a and b)**, which had been present for over 14 months. Early post-operative photograph of free gracilis muscle flap and an unmeshed split-thickness skin graft **(c).** The final reconstructive outcome at 14 months post-op **(d-g).**

Twenty-three patients were ≥60 years old with a mean age of 69 years (range 60-84 years). Twenty-two (96%) had tibial osteomyelitis. Twenty-one patients (91%) were reconstructed with a free gracilis flap (Figure 1) and two patients (9%) required a free latissimus dorsi flap.

The older and younger cohorts had similar distributions of osteomyelitis and C-M staging. As expected, there was a greater proportion of ASA grade III patients in the ≥60 years old group compared to the younger group. Table 4 summarises the range of C-M stages and ASA scores for the groups.

Our overall results are summarised in Table 5. We directly compare outcomes in the young and the old cohorts and identify any statistically significant differences.

### Length of procedure & Length of Stay

**Figure 2:**
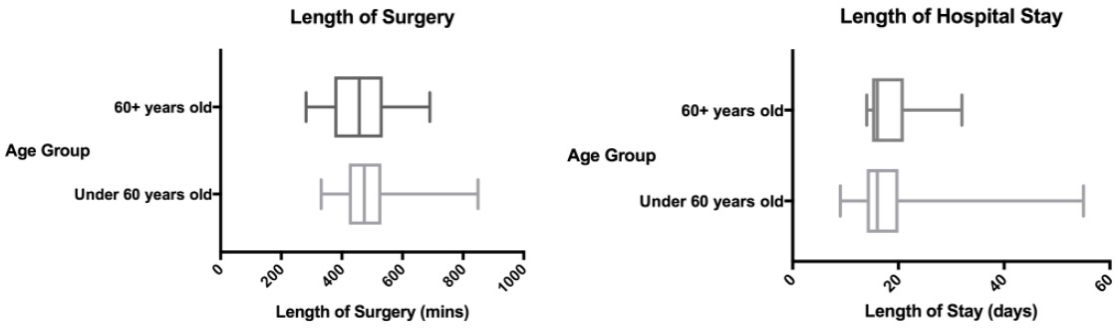
Length of procedure and length of stay.

The overall mean operative time was 489 minutes (range 282-849 minutes) - 466 minutes (range 282-690 minutes) in the older group was and 496 minutes (range 332-849 minutes) in the younger group. There was no statistically significant difference. The mean length of stay was 19 days in both the young and the old cohorts. The length of surgery and length of stay results are summarised in Figure 2.

### Medical Complications

There were seven significant medical complications - five in the younger group (6.9%) and two in the older group (8.7%), although there was no statistical difference. A 45 year-old patient was transferred to the Intensive Care Unit (ICU) with early flap failure and overwhelming sepsis and unfortunately died on post-operative day eight. A 54 year-old patient required transfer to ICU for respiratory support after a prolonged procedure (849 minutes) and airway difficulties. A fit and healthy 40 year-old patient required a brief period of ICU support post-operatively following a transfusion reaction. Two patients in the younger cohort developed antibiotic-related reactions requiring later hospital re-admission. A 62 year-old patient developed an early post-operative pulmonary embolism (despite chemical prophylaxis) and a 61 years-old patient developed pneumonia.

### Surgical Complications

All nine treatment failures required further surgery - eight patients who developed a recurrence of osteomyelitis required further surgery to treat this and one patient required a subsequent amputation for a Marjolin's ulcer (squamous cell carcinoma directly related to the chronic inflammation caused by the underlying osteomyelitis). Excluding planned further surgery and surgery for treatment failures, the overall number of patients who underwent additional unplanned operations (plastic surgical, orthopaedic or both) was 21 (22%). In the younger cohort 20 patients (28%) required further unplanned surgery, whilst in the older group only one patient (4.3%) required further unplanned surgery. This difference was statistically significant (*p*=0.01174).

**Figure 3:**
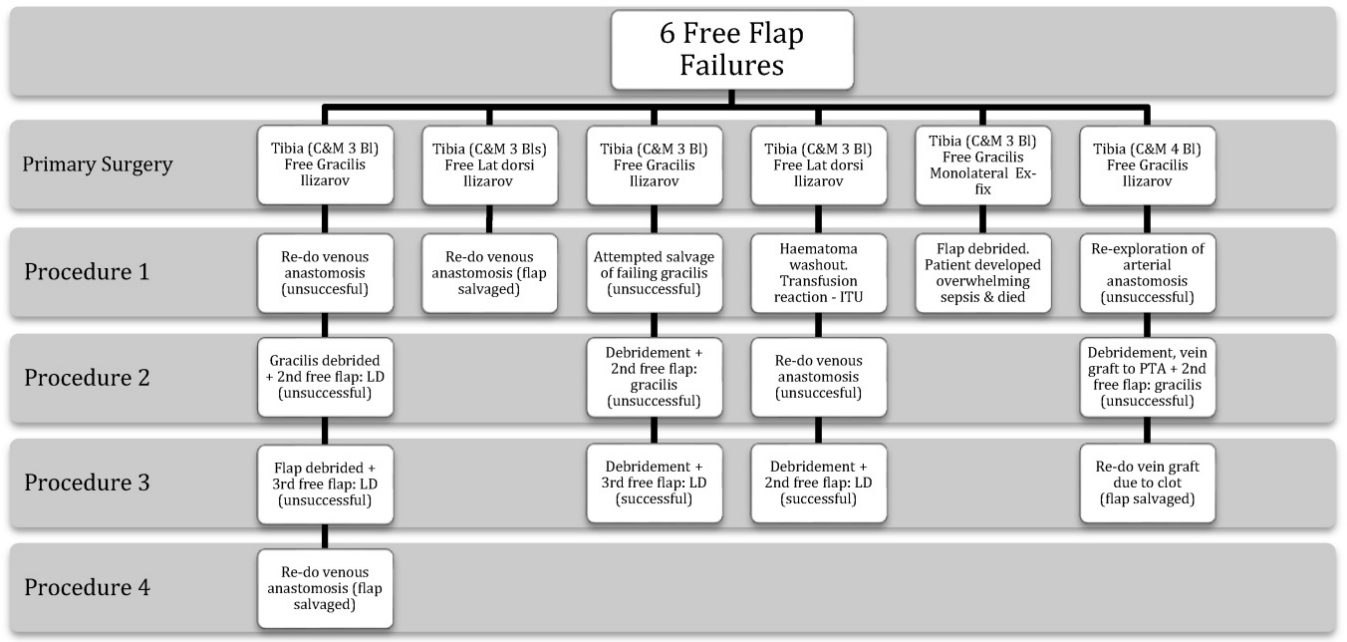
The individualised sequence of events for the six patients who developed primary free flap compromise.

Nine patients required further procedures for plastic surgery-related complications, including six patients who underwent re-operation for primary free flap compromise. Figure 3 illustrates the sequence of additional operations required for these six patients. One compromised primary free flap was successfully salvaged. No attempt at free flap salvage was made in the patient with sepsis who ultimately died, although the flap was removed prior to ICU admission. Four primary flaps were not salvageable and required a second free flap, two of which subsequently failed again requiring a third free flap. Of the five flap failures where salvage was attempted, all patients ultimately had a viable free muscle flap providing definitive soft tissue coverage.

The overall primary free flap failure rate was 5.3%. Although the flap failure rate was 6.9% in the younger cohort compared with no free flap failures in the older cohort, this was not a statistically significant difference.

In this series, one patient required further split-thickness skin grafting to a muscle flap, another patient required debridement of partial muscle necrosis (twice) with further split-thickness skin grafting, a third patient required scar revision of the gracilis donor site and thinning of their flap. Further complications managed conservatively included a donor site stitch abscess, a donor site haematoma and three cases of superficial ulceration or partial muscle flap necrosis.

Seventeen patients required further unplanned orthopaedic procedures. These related to docking site procedures after bone transport, delayed-/non-union, deformity correction, premature union of a corticotomy, fracture or insufficient construct stability. All, apart from one case, occurred in the younger age group. Plastic surgeons were involved in these cases if flap elevation was required.

### Eradication of Infection

Ninety-four patients were followed up for a mean follow-up period of 42 months (range 11-131 months). There were eight recurrences of osteomyelitis (8.5%). There were seven recurrences (9.9%) in the younger group (all tibial osteomyelitis) and one recurrence (4.3%) in the older cohort (femoral osteomyelitis). There was no statistically significant difference in recurrence rates between the old and the young groups (*p*=0.27874).

## Discussion

In the UK, the elderly population is increasing at a dramatic rate. The overall population size is growing and concurrently the proportion of older people is increasing. In mid-2014, 23% of the UK population of 64.6 million people were aged 60 years and over (14.9 million people). By mid-2039, this is predicted to rise to 30% of 74.3 million people (21.9 million people)^14^ - an estimated 47% increase in the population aged 60 years and over in a 25 year period. As the older population increases so will the demand for more definitive and complex treatment of their surgical problems.

There is often a perceived barrier to complex surgery being undertaken in the elderly. Greater co-morbidities and a relative lack of physiological reserve due to aging could lead to the perception of an increased incidence of perioperative morbidity and mortality^4^. However, if anything, our study found the reverse to be true. Our only perioperative mortality was in the younger cohort and a patient in the older group had statistically less chance of requiring further unplanned surgery. Perhaps, the reluctance to undertake complex reconstructive surgery in the elderly is due to concern that operation times will be longer or that medical complications and length of stay are increased^15^? Again, our series did not support these perceptions.

Whilst our study does not investigate those patients who presented with chronic osteomyelitis and did not undergo limb salvage, this number is relatively small (approximately 2% of our referrals) and is usually due to patient choice or poor quality vessels which are inadequate for microvascular surgery. The condition of the limb vasculature is just as likely to be compromised in young intravenous drug users as elderly smokers. Whilst we do not know exact numbers of patients excluded from limb salvage in either age group, this is highly unlikely to have introduced a significant selection bias as the relative numbers are so small. Our decision to undertake orthoplastic limb reconstruction for chronic osteomyelitis is multifactorial, however, if a patient is fit enough for an operation (a relatively low bar with epidural), has vessels that are suitable for free tissue transfer and wants to undergo curative limb reconstruction surgery, then the procedure is offered. The chronicity of the osteomyelitis, number of previous procedures and age of the patient are not determining factors in our decision-making for this group of patients.

Improvements in surgical technique, anaesthesia and perioperative care have significantly improved perioperative mortality rates. Our overall mortality rate was 1.1% (*n*=1). There were seven significant medical complications (7.4%) - two (8.7%) in the older group and five (6.9%) in the younger group. One might postulate a patient selection bias, i.e. only the fit and healthy elderly patients with less extensive chronic osteomyelitis were selected for reconstructive limb salvage. However, the C-M staging for the two cohorts are largely similar and the elderly group had a greater proportion of ASA grade III patients, confirming a higher level of comorbidity in this group.

Many cases of osteomyelitis can be surgically managed without the need for microvascular free tissue transfer. This study only included those patients with chronic osteomyelitis with associated major soft tissue involvement requiring free soft tissue transfer and hence represents the more complex end of chronic osteomyelitis treatment. One might therefore expect a greater proportion of local soft tissue complications and greater recurrence and non-union rates.

Twenty-one of our 95 patients (22%) required unplanned secondary procedures. Nineteen unplanned operations were undertaken by the plastic surgeons in nine patients. There were six compromised primary free flaps in our 95 patients. One was successfully salvaged, so the overall primary free flap failure rate was therefore 5.3%. Four reconstructions were rescued with revision free flap reconstructions. The partial flap necrosis rate was low (3.2%), as we used free muscle flaps (with low rates of tip necrosis) and split-thickness skin grafting. Only one free muscle flap required subsequent thinning. No flap failures were encountered in the elderly group. An overall free flap success rate of 95% is slightly lower than that quoted for some other pathologies requiring elective microvascular free tissue transfer. However, free tissue transfer in chronic osteomyelitis is made more difficult by previous trauma and multiple previous surgeries to the limb in addition to the fibrosis elicited as a result of chronic infection.

Our data demonstrating 100% flap success in osteomyelitis reconstruction in the older cohort is in keeping with the finding that free flap success rates are not influenced by age alone^4^. Previous series' of free flap reconstructions in the elderly for a range of indications are presented in Table 6. To our knowledge this is the first reported series of elderly patients undergoing such reconstructions for treatment of chronic osteomyelitis.

Eight of our patients (8.5%) had a recurrence of osteomyelitis at a mean follow-up of 42 months. Seven were aged under 60 years (9.9%) and one was over 60 years old (4.3%). This overall recurrence rate is slightly lower than similar cohorts previously reported. Cierny and Mader reported a 14% failure rate in all stages of chronic osteomyelitis^5^. Anthony et al. had an 11%^28^ recurrence rate, Tulner 9%^29^ and McNally *et al*. 8.1%^30^ in cohorts where only a proportion of the patients required free flap reconstruction. Tulner *et al.* reported a flap failure rate of 8% confirming the difficult nature of the recipient vessels in these patients^29^.

The success of treating chronic osteomyelitis in patients requiring free flap reconstruction does not appear to be related to age, but may be associated with availability and quality of recipient vessels and blood flow within the affected limb.

## Conclusion

Our study found no evidence that curative surgery for chronic osteomyelitis with simultaneous orthoplastic reconstruction including free soft tissue transfer was less safe or had worse outcomes in those aged ≥60 years compared to a younger cohort. This older group tended towards more favourable outcomes when looking at length of operation, flap failure rate and infection recurrence. Significantly fewer patients underwent unplanned re-operation in the older cohort compared to the younger group. This is unlikely to be due to a selection bias given the older cohort have a greater proportion of ASA grade III patients than the younger group with similar C-M staging in both groups.

In our opinion, both the young and old enjoy satisfactory outcomes from surgical resection of chronic osteomyelitis with simultaneous orthoplastic reconstruction including free-tissue transfer. Age alone should not be a barrier to potentially curative surgical treatment.

## Figures and Tables

**Table 1:** Data fields collected from electronic patient records.

Date of definitive surgery	Length of procedure
Age at definitive surgery	Length of hospital stay
Bone affected	Medical complications
Cierny-Mader anatomic type	Surgical complications
Cierny-Mader physiological class	Further operative procedures required
ASA grade	Free flap failure
Bone resection & stabilisation	Recurrence/Treatment failure
Free flap type	Length of follow-up

**Table 2:** Conditions associated with a compromised host status in the treatment of osteomyelitis.

Local factors in the limb (Bl-host)	Systemic factors (Bs-host)
Arterial ischaemia	Malnutrition
Venous insufficiency	Diabetes
Previous surgery	Smoking
Deep vein thrombosis	Intravenous drug abuse
Lymphoedema	Hypoxia
Radiation fibrosis	Renal/liver failure
Tissue scarring	Immuno-suppression
Retained foreign material/implants	Malignancy
Osteoporosis	Sickle-cell disease
Compartment syndrome	Drug allergies
Obesity	Mental illness

**Table 3:** Osteomyelitis free flap cases by involved bone.

Bone Affected by Chronic Osteomyelitis	Frequency (%)
Tibia	88 (93%)
Femur	1 (1%)
Fibula	2 (2%)
Calcaneum	2 (2%)
Ulna	1 (1%)
Humerus	1 (1%)

**Table 4:** Cierny-Mader (C-M) Staging and ASA Grades for the <60 years and ≥60 years old age groups.

	n	C-M Anatomic Type - frequency (%)				C-M Physiological Group - frequency (%)			ASA Grade - frequency (%)		
		I	II	III	IV	A	B_l_	B_ls_	I	II	III
<60 yrs old	72	0	8 (11%)	51 (71%)	13 (18%)	0	49 (68%)	23 (32%)	31 (43%)	36 (50%)	5 (7%)
≥60 yrs old	23	0	4 (17%)	14 (61%)	5 (22%)	0	14 (61%)	9 (39%)	6 (26%)	11 (48%)	6 (26%)

**Table 5:** Summary Table of Results.

		All Patients (*n*=95)	<60 years old Group (*n*=72)	≥60 years old Group (*n*=23)	*p*-Value
Age in years - mean (range)		49 (18-84)	42 (18-59)	69 (60-84)	-
Length of Follow-up in months - mean (range)^*^		42 (11-131)	42 (11-131)	42 (12-104)	-

Length of Procedure in minutes - mean (range)		489 (282-849)	496 (332-849)	466 (282-690)	0.4249
Length of Stay in days - mean (range)^*^		19 (9-55)	19 (9-55)	19 (14-32)	0.1566

Frequency of Medical Complications (%)		7 (7.4%)	5 (6.9%)	2 (8.7%)	0.32035

**Treatment Failures**					
	Frequency of Treatment Failures (%)^*^	9 (9.6%)	7 (9.9%)	2 (8.7%)	0.31632
	Frequency of Recurrence of Osteomyelitis (%)^*^	8 (8.5%)	7 (9.9%)	1 (4.3%)	0.27874
	Frequency of Related Amputation (%)^*^	1 (1.1%)	0 (0%)	1 (4.3%)	0.24468
	
**Surgical Complications**					
**Flap Failure**					
	Frequency of Primary Free Flap Failure (%)	5 (5.3%)	5 (6.9%)	0 (0%)	0.24148
	Frequency of Primary Free Flap Compromise (%)	6 (6.3%)	6 (8.3%)	0 (0%)	0.17977
	Frequency of Primary Free Flap Salvage (%)	1 (1.1%)	1 (1.4%)	N/A	-
	Frequency of Partial Free Flap Compromise (%)	3 (3.2%)	2 (2.8%)	1 (4.3%)	0.42472
	
**Further Unplanned Surgery^§^**					
	Frequency of Patients requiring further unplanned surgery (%)^§^	21 (22%)	20 (28%)	1 (4.3%)	0.01174

**Table 6:** Review of published series of free flap transfers in older patients.

Reference	Age Group	Free Flap Location/ Indication	No. of Patients (Flaps)	Primary Free Flap Survival (%)	Medical Complications (%)	Surgical Complications (%)	Peri-op Mortality (%)
Özkan et al.^16^ (2005)	≥50	Mainly Head & Neck and Lower Limb	55 (58)	98	25	19	6
MacLeod & Cleland^17^ (1994)	>50	Mixed - mainly Head & Neck Cancer	101	90	-	11	-
Shestak & Jones^18^ (1991)	≥50	Mixed - mainly Head & Neck	92 (94)	99	14	15	5
Peters & Grotting^19^ (1989)	≥60	Head & Neck Malignancy	6	100	-	17	0
Bonawitz et al.^20^ (1991)	≥60	Mixed - mainly Head and Neck Malignancy and Non-Healing Wounds	47 (54)	83	6	19 (major)	2
						34 (minor)	
Reece et al.^21^ (1994)	>65	Cancer	130	95	22	45	2
Serletti et al.^22^ (2000)	≥65	Mixed - mainly Head & Neck and Lower Limb	100 (104)	97	54	28	3
Chik et al.^23^ (1992)	≥65	Mixed - mainly Head and Neck Malignancy and Chronic Osteomyelitis	31 (33)	94	35	65	0
Sosin et al.^10^ (2015)	≥70	Scalp Reconstruction	8	100	-	75	0
Shaari et al.^24^ (1998)	≥70	Head & Neck	52 (56)	100	11	37	6
Beausang et al.^15^ (2003)	>70	Head & Neck	53	96	19	26	4
Malata et al.^25^ (1996)	≥70	Head & Neck Cancer	33 (39)	95	9	33	3
Furnas et al.^26^ (1991)	>70	Mixed - mainly Lower Limb	10	90	-	50	0
Howard et al.^4^ (2005)	≥70	Mainly Head & Neck	197	97	16	28	9
Klein et al.^9^ (2016)	≥78	Mainly Head & Neck	25 (27)	89	70	41	4
Blackwell et al.^27^ (2002)	≥80	Head & Neck Cancer	13	100	62	8	0
Kendall et al. (2018)	≥60	Chronic Osteomyelitis	23	100	9	4	0
